# Editorial: The role of inflammation in neurodegenerative and psychiatric disorders

**DOI:** 10.3389/fncel.2025.1574274

**Published:** 2025-03-11

**Authors:** Dirk M. Hermann, Mingyue Zhang, Anfei Huang, Zenghui Teng

**Affiliations:** ^1^Department of Neurology, University Hospital Essen, University of Duisburg-Essen, Essen, Germany; ^2^Lab for Molecular Neuroscience, Clinic for Mental Health, University of Münster, Münster, Germany; ^3^Lehrstuhl für Systemimmunologie I, Julius Maximilian University of Würzburg, Würzburg, Germany; ^4^Institute of Neuro- and Sensory Physiology, Heinrich-Heine-University Düsseldorf, Düsseldorf, Germany

**Keywords:** neuroinflammation, neurodegenerative disorders, psychiatric disorders, ischemic stroke, Alzheimer's disease, glia cells, immunosurveillance

Inflammation plays a pivotal role in the pathogenesis of neurodegenerative disorders, such as ischemic stroke and Alzheimer's disease, and psychiatric disorders, namely anxiety and depression. Neuroinflammatory responses contribute to neuronal loss in ischemic stroke and neurodegenerative diseases (Neumann et al., 2015; van der Flier and Heneka, 2025; Iadecola et al., 2020). In ischemic stroke, fatigue, anxiety and depression have been described as part of cognitive deficits and sickness behavior, which unlike focal neurological deficits are strongly dependent on cytokine levels (Roth et al., 2021). Subtle neuroinflammation associated with the activation of the indoleamine-2,3 dioxygenase-1 pathway is a central hallmark of major depressive disorder (Hoyo-Becerra et al., 2014). Brain inflammatory responses predispose to oxidative stress, neurotransmitter dysbalance, growth outgrowth disturbances, and synaptic plasticity deficits in neurodegenerative disorders (van der Flier and Heneka, 2025; Iadecola et al., 2020) and psychiatric conditions (Hoyo-Becerra et al., 2014). Targeting neuroinflammatory responses thus may represent a promising way for promoting outcomes in both disease groups.

Neurodegenerative and psychiatric diseases impose a significant health burden, especially on older individuals. The association between neurodegenerative diseases that develop later in life and psychiatric issues that often arise during adolescence or middle age is supported by epidemiological studies (Huang et al., 2024). Experimental studies have pointed out associations of brain immune surveillance disturbances and neurodegenerative disease pathogenesis encouraging clinical treatment trials (Heneka et al., 2024; Scheltens et al., 2021). Given the intricate biology underlying psychiatric and neurodegenerative disorders, there are still numerous critical questions that demand attention. It is vital to elucidate the underlying neuroinflammatory mechanisms to enable the development of new treatment strategies. Therefore, the objective of this Research Topic was to further delve into the role of inflammation in neurodegenerative and psychiatric diseases, aiming to uncover disease mechanisms that may be therapeutically be targeted for enhancing disease outcomes.

This Research Topic published four original research papers, which illustrate current research activities in the neuroinflammation, neurodegeneration and depression fields in an exemplary way. Brain-invading polymorphonuclear neutrophils and lymphocytes play key roles in the development of ischemic injury (Neumann et al., 2015; Herz et al., 2015). The neutrophil-to-lymphocyte ratio (NLR), platelet-to-lymphocyte ratio (PLR), and lymphocyte-to-monocyte ratio (LMR) in peripheral blood was previously shown to be associated with ischemic stroke and stroke outcome (Kim et al., 2018; Zhang et al., 2023), and NLR has specifically been associated with delirium after ischemic stroke (Guldolf et al., 2021). In the research article by Wang et al., who evaluated 1,436 ischemic stroke patients without dementia of the Medical Information Mart for Intensive Care (MIMIC)-IV database requiring intensive care unit (ICU) admission, 214 patients (14.9%) were found to have delirium. In a multivariate logistic regression analysis, the patients in the highest quartile of the NLR (odds ratio [OR] 2.080, 95% confidence interval [CI], 1.282–3.375) and LMR (OR 0.503, 95% CI 0.317–0.798) and the patients in the second quartile of the PLR (OR 1.574, 95% CI 1.019–2.431) were significantly associated with delirium (Wang et al.). A restricted cubic spline function showed a progressive increase in the risk of delirium with higher NLR and PLR and lower LMR, while in a Mendelian randomization analysis only the PLR was negatively associated with delirium risk (Wang et al.). The data underline the relevance of blood leukocyte markers for cognitive and memory disturbances. Future studies will have to define the underlying molecular mechanisms.

Oxidative stress is a major contributor to inflammatory cell damage, specifically via oxidation of lipid membranes. Lipoxygenases (LOXs) are a family of lipid-oxidizing enzymes, which generate eicosanoids and related compounds from arachidonic acid and other polyunsaturated fatty acids (Jin et al., 2008). The 12/15-LOX is special in that it can directly oxidize lipid membranes containing polyunsaturated fatty acids, without the preceding action of a phospholipase, leading to the direct attack on organelles (van Leyen et al., 2014). This presumably underlies the cytotoxic activity of 12/15-LOX, which is upregulated in neurons and endothelial cells after stroke (van Leyen et al., 2014). In their original research article, Cakir-Aktas et al. exposed mice to transient proximal middle cerebral artery occlusion (MCAo) and evaluated the effect of the 12/15-LOX inhibitor ML351 (50 mg/kg) on ischemic brain damage. Infarct volume, neurological deficits, lipid peroxidation, and pro-inflammatory cytokine levels (interleukin-1β, interleukin-6, tumor necrosis factor-α) were significantly attenuated by 12/15-LOX inhibition, as was NOD-like receptor protein (NLRP)-1 and−3 signaling in neurons and non-neuronal cells (Cakir-Aktas et al.). These results suggest that 12/15-LOX inhibition suppresses ischemia-induced inflammation in the acute and subacute phases of stroke by suppressing inflammasome activation.

Via tripartite synapses, astrocytes play critical roles in synaptic transmission (Dzyubenko and Hermann, 2023). Astrocytes have been involved in synaptic dysfunction in Alzheimer's disease (AD) (Masliah et al., 1996). AD patients have imbalanced cholesterol metabolism, demonstrated by high levels of side-chain oxidized cholesterol known as 27-hydroxycholesterol (27-OH), which can abolish synaptic connectivity during neuronal maturation. In their original research article, Spanos et al. reported a downregulation of glutamate transporters together with increased glial fibrillary acidic protein in the hippocampus of CYP27Tg mice, a mouse model of brain oxysterol dysbalance. Glutamate transporter-1 (GLT-1) downregulation was also observed when wildtype mice were fed with high-cholesterol diets. To study the relationship between astrocytes and neurons, a 3D co-culture system was used, which reproduced the effects of 27-OH previously observed in 2D neurons and *in vivo* (Spanos et al.). Moreover, the authors found novel degenerative effects of 27-OH in astrocytes that did not appear in 2D cultures, together with the downregulation of GLT-1 and glutamate-aspartate transporter (GLAST) (Spanos et al.). The authors proposed that this transporter dysregulation leads to neuronal hyperexcitability and synaptic dysfunction. Taken together, these results report a new mechanism linking oxysterol imbalance and synaptic dysfunction through effects on astrocyte function.

GATA1 is a member of the GATA transcription factor family and a critical factor in hematopoietic system development (Gao et al., 2015). GATA1 has previously been shown to be increased in the dorsolateral prefrontal cortex of patients suffering from depression, where it was found to act as a transcriptional repressor of synapse-related genes (Kang et al., 2012). Building upon these earlier works, Choi et al. in their original research article investigated how GATA1 globally altered gene expression using multi-omics approaches. Through the combined analyses of ChIPseq, mRNAseq, and small RNAseq, the authors profiled genes that are potentially affected by GATA1 in cultured cortical neurons, and Gene Ontology analysis revealed that GATA1 might be associated with immune-related functions (Choi et al.). Hypothesizing that GATA1 induces immune activation, which has detrimental effects including synapse loss and depressive-like behavior, the authors first performed a microglial morphometric analysis in GATA1 overexpressing brains. Fractal analysis showed that the ramification and process length of microglia decreased in brains exhibiting GATA1 overexpression, suggesting that GATA1 increases microglial activation (Choi et al.). Through flow cytometry and immunohistochemical analysis, the authors found that activated microglia showed pro-inflammatory phenotypes characterized by CD86 and CD68 expression (Choi et al.). Finally, they demonstrated that the effects of GATA1 overexpression including synapse loss and depressive-like behavior could be blocked by inhibiting microglial activation (Choi et al.). These results show that microglial activation is centrally involved in GATA1′s effects on neuronal plasticity and depressive-like behavior.

This Research Topic provides four take home messages: (1) Peripheral immune responses, namely neutrophil, lymphocyte and platelet counts, are valuable predictors of cognitive and memory disturbances in ischemic stroke patients that allow the identification of patients developing delirium. (2) Lipoxygenases, namely 12/15-LOX, contribute to post-ischemic oxidative brain damage via NLRP-1 and−3-dependent inflammasome activation. (3) The side-chain oxidized cholesterol 27-OH decreases glutamate transporters GLT1 and GLAST in astrocytes, which evokes neuronal hyperexcitability and synaptic dysfunction in 3D neuronal-astrocyte co-cultures. (4) The transcriptional repressor of synapse-related genes GATA1 induces microglial activation that induces synaptic plasticity disturbances and depressive-like behavior. Together, these papers emphasize the interplay of diverse cell types (neurons, astrocytes, microglia, and leukocytes) in the degenerating and psychiatric brain, which maintain inflammatory responses, predispose to oxidative injury, most notably of membrane lipid structures, and disturb synaptic integrity and function. With respect to these processes, neurodegenerative and psychiatric diseases share a surprisingly large number of disease mechanisms. Future studies will have to show if treatments suitable in one disease area may have similar application in another one. This might strongly foster clinical translation in the neurodegeneration and psychiatry fields.
